# Evaluation of Conditions to Improve Biomass Production by Submerged Culture of *Ganoderma* sp.

**DOI:** 10.3390/microorganisms10071404

**Published:** 2022-07-12

**Authors:** Catalina Rosales-López, Alejandro Vargas-López, Mariana Monge-Artavia, Miguel Rojas-Chaves

**Affiliations:** Centro de Investigación en Biotecnología, Instituto Tecnológico de Costa Rica (ITCR), Cartago 159-7050, Costa Rica; alevalo.1392@gmail.com (A.V.-L.); marige97@gmail.com (M.M.-A.); mirojas@itcr.ac.cr (M.R.-C.)

**Keywords:** *Ganoderma*, culture medium, bioreactor, biomass, inoculum, secondary metabolites

## Abstract

In the present investigation, the conditions for in vitro submerged culture of a native strain of *Ganoderma* sp. were evaluated. Different culture medium ingredients, inoculum concentrations, inoculation methods, configuration, and airflows were evaluated to improve biomass production. The addition of thiamine and olive oil to the culture medium increased biomass production, as well as inoculating 6.6 g/L since there are no significant differences in biomass growth according to inoculum origin (pre-inoculum, discs or with spores). The best configuration of the 3 L stirred tank bioreactor was using three impellers and a porous air diffuser of 0.25 volume per volume per minute (vvm), the dry biomass concentration was 22.6 g/L after 12 days of cultivation at 30 °C, much higher than other investigations. This study provides relevant information for pilot-scale production of this fungus for future secondary metabolites. The culture medium was optimized, and it was defined that the concentration and origin of the inoculum did not influence the growth of Biomass, but the aeration and the configuration of the system allowed the establishment of protocols for the cultivation of *Ganoderma* sp.

## 1. Introduction

The genus *Ganoderma* contains many large species of bracket fungi belonging to the phylum Basidiomycota. Most of the species are saprophytes and grow on decomposing trunks [1]. Over 250 species of *Ganoderma* have been described worldwide, most of them classified based on pleomorphic characteristics [2]. In Costa Rica, nine species of this genus have been identified morphologically: *G. australe*, *G. amazonense*, *G. dorsale*, *G. longistipitatum*, *G. oerstedii*, *G. orbiforme*, *G. perzonatum*, *G. resinaceum*, and *G. stipitatum* [3,4]. The main taxonomic characteristic of this genus is the double-walled basidiospore. The inner wall is thick and yellowish-brown with numerous endosporic projections, and the external wall is thin, smooth, and hyaline [5]. Further characterization of *Ganoderma* is based on its microstructures; a molecular analysis is difficult because of the lack of gene sequences from neotropical collection zones.

*Ganoderma* is recognized for its medicinal value. It is used to treat diseases, including gastric ulcers, chronic hepatitis, hypertension, nephritis, asthma, arthritis, bronchitis, insomnia, cancer, diabetes, and anorexia [6]. Among the many bioactive compounds present are steroids, lignins, lectins, ganomycins, vitamins, nucleosides, nucleotides, alkaloids, amino acids, polysaccharides, and triterpenes [7]. Special attention has been given to the last two groups of compounds mentioned due to their effects on conditions relevant to human health.

Ganoderic acids are a group of oxygenated triterpenes derived from lanosterol, which have been isolated from fruiting bodies, mycelium, and spores of *Ganoderma* sp. This family of compounds is numerous due to the possible substitutions on carbons 3, 7, 11, 12, 15, 22, 23, 24, and 25. Pharmacological effects that have been tested for these molecules include cytotoxicity against hepatic cancer cells, cholesterol-synthesis inhibition, antihistaminic activity, α-glucosidase inhibition, and antihypertensive activity [7]. 

On the other hand, the most important polysaccharides produced by *Ganoderma* sp. are β-D-glucans, which are constituted mainly by glucose monomers linked by β-1,3 bonds. Ramifications on the structures are made by β-1,6 bonds. These molecules have been isolated from fruiting bodies, mycelium, and spores, but also in spent culture media. Previous studies have shown the following effects for these polysaccharides: immunomodulatory activity, antioxidant capacity, anti-inflammatory effects, hepatoprotective activity, and triglyceride reduction effects [6]. 

In submerged cultures, both ganoderic acids and polysaccharides can be obtained. The production of these compounds depends on the biological and physicochemical characteristics of the fermentation, such as the culture medium used, agitation, temperature, and pH, among others [1,6,7]. In recent years, there have been attempts to increase the production of these bioactive compounds in vitro using biotechnology. As a first stage, the fungus is grown in an artificial culture medium under controlled conditions [8].

The objective of this research was to evaluate different components of the culture medium and to define the optimum inoculum density and inoculation technique to maximize biomass production of *Ganoderma* sp. in flasks and bioreactors using a strain collected in the Central Valley of Costa Rica.

## 2. Materials and Methods

The trials were carried out at the Instituto Tecnológico de Costa Rica, at the Centro de Investigación Biotecnología, (CIB).

### 2.1. Source and Maintenance of the Strain of Ganoderma sp.

The strain of *Ganoderma* used in this study was identified molecularly and morphologically as *Ganoderma curtisii* and was provided by the Fungal Collection of the Centro de Investigación en Innovación Forestal (CIF) at the Instituto Tecnológico de Costa Rica (accession number CIIBI-007A, biodiversity permit R-CM-ITCR-005-2019-OT). The strain was grown on potato dextrose agar (PDA) (CM-0139, OXOID) in 10 cm Petri plates in an incubator (Digisystem) at 30 °C for 8 days and subcultured weekly by transferring segments of mycelium and culturing under the same conditions described.

### 2.2. Optimization of Culture Medium

The effects of olive oil, calcium and copper salts, and thiamine (vitamin B1) on *Ganoderma* sp. biomass concentration in a liquid medium were evaluated using a three-factor completely randomized factorial design, with two levels (presence or absence) of each of the three factors, and with three replicates per treatment combination (Table 1). Olive oil, calcium and copper salts (as a single factor), and thiamine, were added to a base medium containing 30.0 g/L glucose, 5.0 g/L peptone, 5.0 g/L yeast extract, 0.5 g/L KH_2_PO_4_, 0.5 g/L K_2_HPO_4_, 0.5 g/L MgSO_4_ 7H_2_O [9,10]. pH was adjusted to 5.5 with 1 M HCl or NaOH before sterilization. Each component of the culture medium was sterilized separately at 121 °C for 20 min, except thiamine, which was filtered through 0.22 µm. Solutions were mixed aseptically after cooling to room temperature.

Experiments were conducted in 250 mL Erlenmeyer flasks. The culture medium (50 mL) was inoculated with three 8 mm disks of biomass grown on PDA for 8 days. Cultures were maintained at room temperature (25 ± 2 °C) in an orbital shaker at 100 rpm for 14 days. The final biomass was recovered by vacuum filtration and dried at 65 °C for 48 h before weighting.

The best culture medium (medium C, Table 2), as defined by the factorial design, was tested against a supplemented medium (C with 1% *v*/*v* olive oil) and a commercial medium (Potato Dextrose Broth, PDB) using a one-way ANOVA. Growth conditions and biomass recovery techniques were the same as described before.

### 2.3. Selection of the Inoculation Method

The effect of the inoculation method on the final biomass of *Ganoderma* sp. In a liquid medium was evaluated. Three methods of inoculation were evaluated in triplicate: mycelial disks from eight-day cultures [13], a spore suspension [14], and the pre-inoculation method. For the spore suspension, 10 mL of sterile water was added to an eight-day culture on a Petri plate. After mixing with the culture, the water was collected and added to a fresh medium in an Erlenmeyer flask. For the pre-inoculation method, mycelia from an eight-day-old flask culture were filtered with a sieve and added to cultures at a concentration of 20 g (fresh weight)/L.

For each experiment, cultures were grown at room temperature (25 ± 2 °C) in 50 mL of medium with agitation at 100 rpm for 14 days. Biomass was recovered after 14 days, and dry weight was measured.

### 2.4. Determination of the Optimum Inoculum Concentration

To determine the best inoculum concentration for maximum growth of the fungus, three inoculum concentrations were tested in triplicate: 6.6 g/L [11], 10.0 g/L, and 20.0 g/L. The pre-inoculation method was used to inoculate each treatment. Cultures were maintained at 100 rpm at room temperature (25 ± 2 °C) for 14 days. The biomass was recovered, and dry weight was determined. A growth index was determined as an additional response variable using the following equation:

(1) $$GI=\frac{X_{f}-X_{0}}{X_{0}}$$

where:

*GI*: growth index (g/g).
*X_f_*: final dry biomass weight (g).
*X*_0_: initial dry biomass weight (g).

### 2.5. Growth Kinetics

After defining the optimum culture medium composition, inoculum type, and inoculum concentration, growth kinetics of *Ganoderma* sp. in a liquid medium were evaluated. Dry biomass concentration was measured by taking three flasks at days 0, 2, 4, 6, 8, 10, 12, and 14. Data were graphed over time to determine the specific growth rate (h^−1^). Mathematical models were fit to the data by non-linear regression and the Levenberg–Marquardt algorithm (tolerance of 1.0 × 10^−8^) using the software CurveExpert Professional version 2.6.5. The growth curve was used to determine when to transfer flask cultures to the bioreactor.

### 2.6. Culture in the Bioreactor

A 3 L (2 L working volume) Applikon Biotechnology bioreactor was used. The culture medium in the bioreactor was stirred by three impellers: marine propeller (lower), pitched-blade turbine (middle), and Rushton turbine (upper). Baffles were not used during fermentations. The bioreactor was inoculated with 10 g/L of fresh biomass from 8-day flask cultures. The initial dissolved oxygen concentration was 100%. Cultures were maintained at 30 °C with 350 rpm agitation for 12 days.

### 2.7. Determination of Airflow Rate

Compressed air (30 psig) was introduced through a porous diffuser. Two airflow rates (0.25 and 1.0 vvm) were tested in duplicate. The volumetric oxygen mass transfer coefficient (kLa) was determined before inoculation using the dynamic method with four replicates. Biomass was collected after 12 days, and dry weight was determined.

### 2.8. Statistical Analyses

All experiments used a completely randomized design. The response variable for all experiments was the dry biomass concentration (g dry weight/L). Analyses were performed in Minitab 19 with a 95% (α = 0.05) confidence level. Equal variances were assumed for all analyses, and the normality of residuals was verified.

## 3. Results

Dry biomass concentration from each medium is shown in Table 2. Biomass concentration was the highest in treatment “C”, which contained olive oil and thiamine. In contrast, biomass dry weight concentration was lowest in treatment “E”, which contained salts and thiamine.

The effects on biomass concentration of olive oil and thiamine were statistically significant, as seen in Table 3 and the interactions between olive oil–thiamine (Table 4) and salts–thiamine (Table 5). Moreover, the dry biomass concentration was higher in cultures grown in a medium with added olive oil and thiamine than in a medium without these components. The interactions between factors show that cultures grown in a medium containing olive oil produced more biomass when thiamine was also added to the medium. In contrast, cultures grown in a medium with added salts (CuSO_4_ and CaCl_2_) produced a lower concentration of biomass when thiamine was also present in the medium.

**Figure 1:**
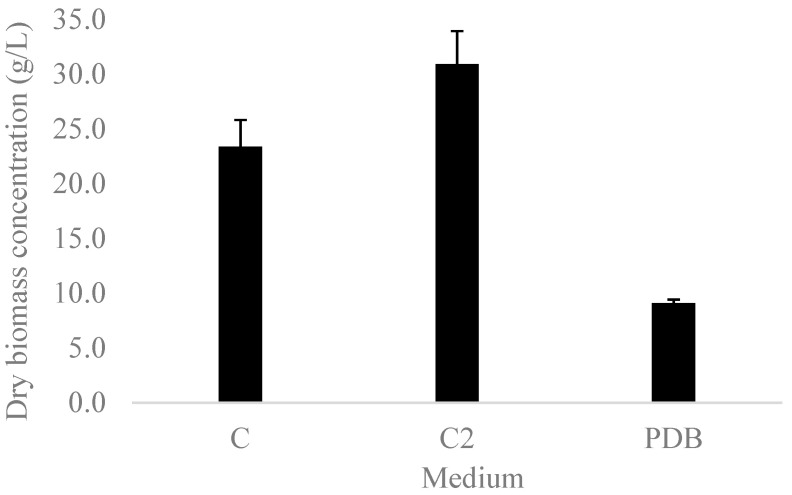
Dry biomass concentration obtained after 14 days in the optimized medium (C), optimized medium supplemented with 9.1 g/L olive oil (C2), and commercial potato dextrose medium (PDB). Error bars indicate the standard deviation of three independent replicates.

Due to the important increase in biomass concentration of *Ganoderma* sp. with the addition of olive oil to the culture medium, the effect of adding oil at 1% *v*/*v* (9.1 g/L) was examined. Figure 1 shows that the growth of the organism was 32% greater in supplemented medium (C2) than in the medium selected in the factorial design (C), and 240% greater than in commercial medium (PDB), according to Tukey’s pairwise comparison (α = 0.05).

### 3.1. Inoculation Method

**Figure 2:**
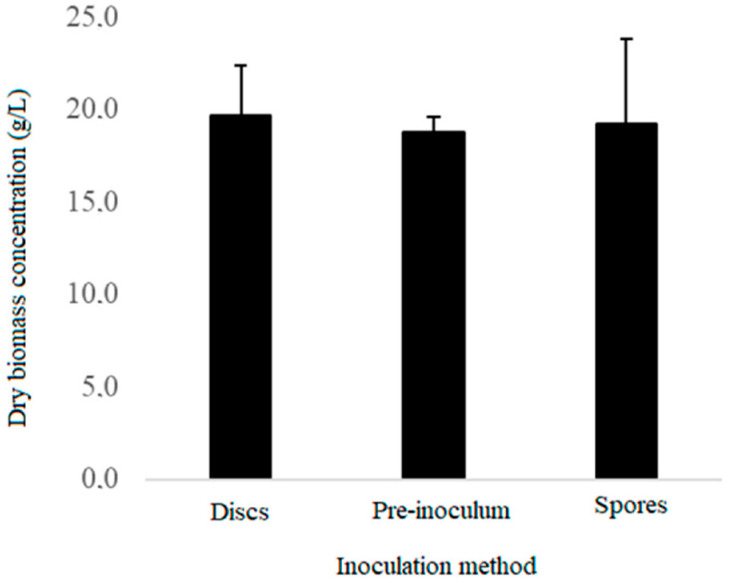
The effect of the inoculum method on the dry biomass weight of *Ganoderma* sp. mycelia.

The pairwise comparison showed no significant differences between the inoculation methods, with a 95% confidence level. Figure 2 shows the effect of the inoculation method on the dry weight of *Ganoderma* sp. mycelia. However, the initial inoculum concentration was different for each method used and may have affected the final dry weight of the mycelium, as demonstrated by [15].

Other studies have shown that the size, homogeneity, and concentration of the inoculum may affect the growth phases of this fungus. Researchers have used different inoculation techniques for *Ganoderma*, but pre-inoculation is the most used [16]. Although biomass production was not significantly different among inoculation methods, pre-inoculation resulted in more homogeneous growth and better distribution and was therefore selected for use in later experiments.

### 3.2. Inoculum Concentration

**Figure 3:**
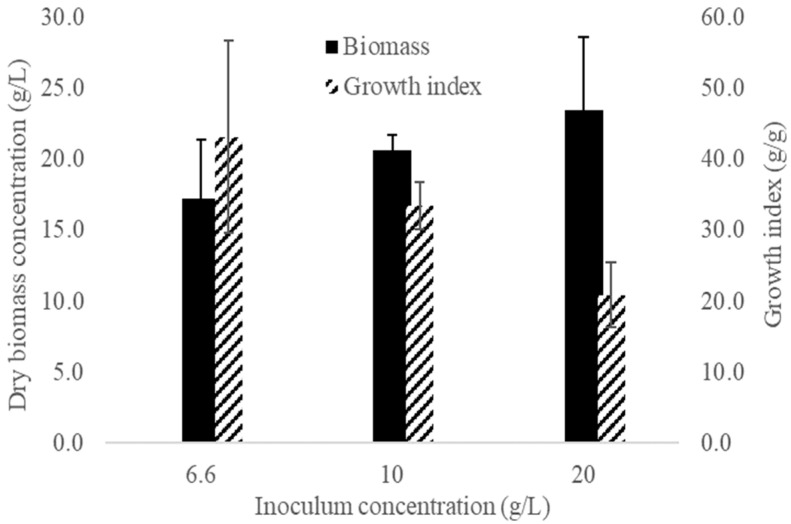
Dry biomass concentration and growth index obtained after 14 days using different inoculum concentrations. Black bars: dry biomass concentration (g/L). Striped bars: growth index (g/g). Error bars indicate the standard deviation of three independent replicates.

Figure 3 shows the effect of the inoculum concentration on the dry biomass concentration and growth index. The dry biomass concentration (black bars) tended to increase with increased inoculum concentration. In contrast, the growth index (striped bars) decreased as the inoculum concentration increased. According to Tukey’s pairwise comparison analysis (α = 0.05), the dry biomass concentration did not differ significantly among the three treatments, while the growth index in the treatment inoculated with 6.6 g/L was significantly higher than in the treatment inoculated with 20.0 g/L.

### 3.3. Growth Kinetics

In Table 6, specific growth rate of *Ganoderma* sp. in the submerged culture obtained from different growth models.

**Figure 4:**
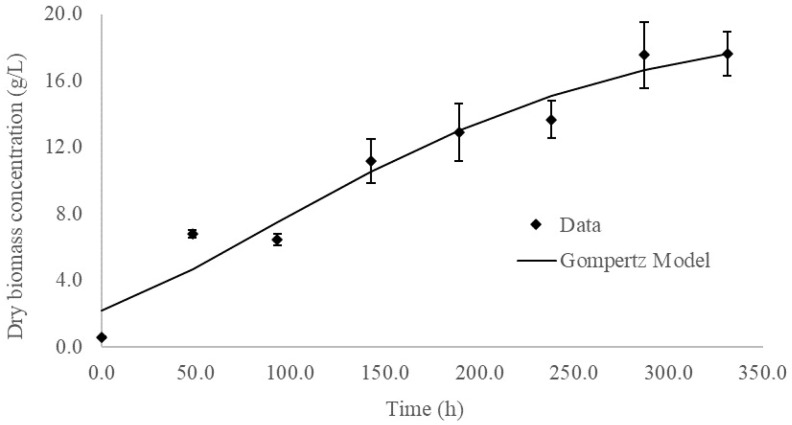
Growth kinetics of the *Ganoderma* sp. in the flask culture. Error bars indicate the standard deviation of three independent replicates.

Using an inoculum concentration of 10 g fresh weight/L, growth kinetics were determined for *Ganoderma* sp. cultures in liquid medium in flasks. The curve of dry biomass concentration over time (Figure 4) showed the absence of an adaptation or latent phase. The beginning of the stationary phase was seen at 300 h (12 days) of growth.

### 3.4. Growth in the Bioreactor Airflow Rate Tests

**Figure 5:**
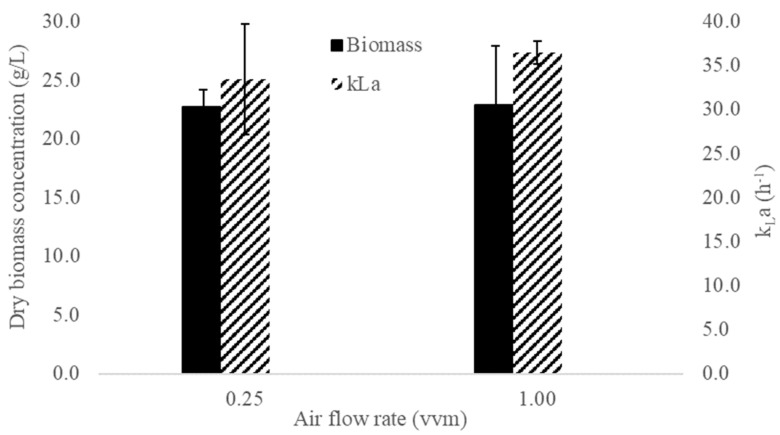
Dry biomass concentration (at 12 days) and k_L_a (before inoculation) for the two airflow rates tested at the stirred tank bioreactor. Error bars indicate the standard deviation of two independent replicates.

Statistical assumptions of the normality of residuals were fulfilled for the experimental designs used in this section. The equal variance assumption was fulfilled for the dry biomass concentration, but not for k_L_a, in which case the data were analyzed by Welch’s ANOVA with the Games–Howell pairwise comparison. Figure 5 shows the dry biomass concentration and k_L_a for the airflow rate experiments in a stirred tank bioreactor with a working volume of 2 L, equipped with a porous air diffuser and three impellers (marine propeller, pitched blade turbine, and Rushton, from the bottom to the top). Dry biomass concentration (black bars) was similar in the two treatments, while k_L_a (striped bars) increased slightly with a greater airflow rate. The pairwise comparison showed that neither of the results was statistically significant (α = 0.05), which suggests that the airflow rate did not exert any significant effect on the growth of the fungus and the mass transfer coefficient.

The effect of the airflow rate on the growth of *Ganoderma* sp. in stirred tank bioreactors has been previously studied. Tang, Y. et al. (2003) [17] obtained a maximum concentration of 15.62 g dry biomass/L after eight days of culture with a k_L_a of 78.2 h^−1^ (0.5 vvm, 200 rpm); however, biomass concentration decreased to 11.3 g/L after 15 days. Moreover, using an airflow rate of 1.0 vvm (96.0 h^−1^), biomass production remained constant at 13.5 g/L after approximately 10 days. The dry biomass concentration obtained in this study was 70% greater than that reported by Wagner R. et al. (2003) [14] using the same airflow rate (relative to the working volume, vvm), but the k_L_a was nearly three times lower (96.0 h^−1^ vs. 36.5 h^−1^).

Although the growth of *Ganoderma* sp. in the bioreactor was not significantly affected by the airflow rate, the dry biomass concentration obtained in this work surpassed that reported in the literature for some batch mode (and even fed-batch mode) fermentations. Table 7 shows the dry biomass concentrations reported in recently published articles and the conditions used.

## 4. Discussion

The positive effect of olive oil on the growth of *Ganoderma* sp. in a liquid medium has been previously reported. Chang, M. et al. (2006) and Yorulmaz, A. et al. (2013) [16,25] found that the addition of olive oil to the culture medium at concentrations of 3.0 g/L and 4.0 g/L, respectively, caused an increase in the final biomass concentration. Yorulmaz, A. et al. (2013) [25] found that the addition of some vegetable oils (including olive oil) and fatty acids, such as oleic acid, the principal component of olive oil, increased biomass concentration by 65% and 130%, respectively. On the other hand, the authors of [26] studied the fatty acid and sterol content of different species of *Ganoderma* and found that oleic acid, palmitic acid, linoleic acid, and stearic acid are the major fatty acids present in the fungal biomass. Considering this, the positive effect of olive oil on the biomass growth of *Ganoderma* sp. might be because it provides certain fatty acids or their precursors required by the organism.

The importance of thiamine as an enzymatic cofactor in the central metabolism of many fungi has been established [12]; however, few authors have studied its effect directly on the growth of the genus *Ganoderma*. Thiamine is routinely added to liquid culture medium used in research on the growth of *Ganoderma* sp., but its requirement as an essential nutrient has not been demonstrated. In the present study, the addition of this vitamin to the culture medium improved biomass production in submerged culture. Jo, W. et al. (2009) [18] also reported positive effects of thiamine on the growth of *G. applanatum* in a semisolid medium at a concentration of 0.1 mg/L.

The effect of interactions between olive oil and thiamine, or inorganic salts (CuSO_4_ and CaCl_2_) and thiamine, on the growth of *Ganoderma* sp. has not been previously reported. Table 4 summarizes the results of Tukey’s pairwise comparison for the olive oil–thiamine interaction. Pairs of treatments sharing the same letter (a, b, or c) were not statistically different from each other. The dry biomass concentration obtained in treatments with high levels of both olive oil and thiamine was significantly higher than in treatments including only one of the two factors. These results suggest a synergy between thiamine and the fatty acids present in olive oil; however, the biochemical explanation is unclear.

In contrast, the interaction between salts and thiamine observed in Table 5 was different. The dry biomass concentration obtained in treatments that included salts and thiamine together was not statistically different from the concentrations obtained in treatments without those components. Moreover, in the treatments with a high level of salts, the addition of thiamine did not change the dry biomass concentration significantly. This suggests an antagonistic interaction between the salts (CuSO_4_ and CaCl_2_) and thiamine. In a recent publication, Schnellbaecher A. et al. (2019) [12] reviewed factors that affect the stability of vitamins in the most common culture medium. They reported that copper salts tend to increase the velocity of thermal degradation of thiamine in a solution, particularly when phosphate buffers are used. As for the influence of the calcium salts, there are no reports of direct interactions between calcium ions in solutions containing thiamine. Therefore, the effects observed in this study were likely due to reactions between thiamine and copper ions.

With respect to the addition of olive oil to the culture medium, the effect of increasing oil concentration to 1% *v*/*v* (9.1 g/L) was examined. The growth of the organism was 32% greater in supplemented medium (C2) and these results coincide with those reported by Fei, Y. et al. (2019) [27] and Wei, Z. et al. (2014) [28] for olive oil concentrations of 1% *v*/*v* and 3% *v*/*v*, respectively. Thus, it is possible to affirm that the C2 medium was adequate for the growth of *Ganoderma* sp. in the submerged culture.

Fang, Tang, and Zhong (2002) [15] studied the influence of inoculum concentration on the growth and morphology of *Ganoderma* sp. in a liquid medium and the effect on the production of polysaccharides and ganoderic acids. These authors reported maximum dry biomass concentrations after eight days of culture. After 14 days, the dry biomass concentrations were estimated to be 9.4, 10.2, 8.1, and 8.6 g/L for cultures inoculated with 1.4, 3.4, 6.6, and 13.4 g/L, respectively.

As seen in Figure 3, the growth index was highest (43.10 g/g) for cultures inoculated with 6.6 g/L (0.33 g/L dry biomass). A higher growth index (133.29 g/g) was obtained by Fang, Tang, and Zhong (2002) [15] for cultures inoculated with 1.4 g/L (0.07 g/L dry biomass). However, the final dry biomass concentration (9.4 g/L) was lower than that obtained in the present study (17.26 g/L).

### 4.1. Growth Kinetics

Predicted values for the specific growth rate for the exponential Gompertz and Richards models were of the same order of magnitude, while the logistic model generated a value of one order of magnitude greater. The mathematical model with the lowest values for the standard error of the regression and corrected Akaike information was selected. The use of these criteria instead of the coefficient of determination (r^2^) to analyze the goodness of fit of nonlinear regression models has been discussed previously [20]. The Gompertz model best described the growth of *Ganoderma* sp. In a liquid medium and predicted a specific growth rate of 0.0087 ± 0.0019 h^−1^. Spiess, A. et al. (2010) [20] used the logistic model to describe the variation in biomass concentration over time. These authors found a specific growth rate of 0.019 h^−1^ for a “wild type” strain in culture conditions similar to those of this study. The growth rate obtained by these authors is higher than that obtained in our study with the Gompertz model (0.0087 h^−1^) but is similar to the estimate from the logistic model (0.0139 h^−1^).

Modeling the growth of the *Ganoderma* sp. culture in liquid medium generates valuable information about the performance of the culture system; however, the physiological meaning of the growth parameters obtained from non-structured and non-segregated models should be interpreted with caution. The growth mechanisms of filamentous fungi in liquid medium are quite different from those of unicellular organisms, such as bacteria or yeasts, and the assumptions used to construct many of the models are not always adequately fulfilled [29,30]. For this reason, it is particularly important to evaluate statistically diverse mathematical models for the description and prediction of biomass growth of filamentous fungi in liquid medium.

### 4.2. Growth in the Bioreactor

Kim, H. et al. (2006) [21] studied the individual effects of agitation speed and airflow rate on the growth of *G. resinaceum* in a 3.0 L (working volume) bioreactor with a single Rushton turbine. The dry biomass concentration obtained after 15 days of culture was greater using an airflow rate of 0.5 vvm than with 1.0 vvm (18.5 g/L vs. 17.9 g/L). In our study, dry biomass concentration after 12 days of growth using an airflow rate of 1.0 vvm (22.8 g/L) was 44% higher than that reported by Tang, Y.-J. (2003) [17] after the same time (15.9 g/L). A possible explanation may be found in the culture medium composition used in the two studies. The medium used by Kim, H.M. (2006) [21] lacked certain components necessary for adequate fungal growth [28], such as KH_2_PO_4_, MgSO_4_ 7H_2_O, and thiamine.

In contrast to the previously mentioned research, in this study, the increased airflow rate did not affect the final dry biomass concentration or the volumetric oxygen mass transfer coefficient. We suggest a possible explanation as follows: increasing the airflow rate did not improve biomass concentration because k_L_a did not improve either, which means that the organism experienced the same oxygen availability in both conditions (0.25 and 1.00 vvm). Since *Ganoderma* sp. is a strictly aerobic fungus, oxygen concentration is a critical parameter for submerged growth and may even be the limiting substrate. On the other hand, we suggest that k_L_a did not improve with a higher airflow rate because the bottom impeller (marine propeller) flooded at higher airflow rates. Salmon, D. et al. (2016) [22] proposed a correlation for the estimation of flooding conditions for various impellers. Using the equation for a down-pumping Lightnin A310 impeller (similar to the one we used), we obtained a critical airflow rate of 0.3 vvm at 350 rpm, which is close to the lower value used in this work (0.25 vvm). This means that beyond 0.3 vvm, the bottom impeller entered the flooding regime, in which gas bubbles were not dispersed correctly, negatively affecting the oxygen transfer rate.

One final aspect to consider is the obstruction of the air diffuser by fungal growth on its surface. When fermentations were concluded, fungal biomass was observed around the diffuser; the mass had a compact consistency and was difficult to remove. It was not possible to determine when the mass formed; however, since oxygen concentration fell to 0% in less than four days (for all fermentations), it probably grew to a critical size during the first days of culture. Moreover, in the final days of the fermentation, the fungal biomass tended to accumulate over the motor shaft and the bioreactor walls and impeded the homogenization of the medium by agitation. This situation has been previously reported for submerged cultures of filamentous fungi and is a problem that affects biomass quantification by sampling and fermentation yield calculations [31].

Growth in this study was lower than that obtained by Tang, Y.-J., and Zhu, L.-W. (2010) [11]; however, these two research groups used a considerably larger amount of substrate than that used in the present study. In contrast, the results of this research are similar to those obtained by Salmon, D. et al. (2016) [22] and Tang, Y.-J.; Zhong, J. [23], using a fed-batch mode of operation. To our knowledge, this is the first report on the use of a porous air diffuser for submerged cultures of *Ganoderma* sp. in stirred tank bioreactors and its effect on the mass transfer coefficient.

## 5. Conclusions

The results of this study showed that the addition of thiamine and olive oil to the culture medium significantly improved biomass production of a strain of *Ganoderma* native to Costa Rica. Although the inoculum concentration and method of inoculation did not influence biomass growth, the study contributes toward the standardization of inoculation protocols for *Ganoderma* sp. Fermentations in stirred tank bioreactors using three impeller types and a porous air diffuser generated 22.6 g/L of dry biomass after 12 days of culture with less air consumption (0.25 vvm). However, the problem of biomass adhesion to the surface of the air diffuser should be resolved in future research. This work will contribute to the establishment of high-density cultures of *Ganoderma* sp. in stirred tank bioreactors for research purposes, as well as for the efficient and cost-effective production of secondary metabolites on a pilot scale.

## Figures and Tables

**Table 1:** Components studied to optimize the culture medium.

Factors		Concentration (g/L)		Reference
		Low (–)	High (+)	
Olive oil		0.00 *	3.00	[9]
Salts	CaCl_2_ 2H_2_O	0.00	1.45	[10]
	CuSO_4_ 5H_2_O	0.00	0.25	[11]
Thiamine		0.00	0.05	[12]

* not added.

**Table 2:** Dry biomass concentrations in treatments evaluated for optimization of the culture medium.

Medium	Variables			Dry Biomass Concentration (g/L)	Tukey’s Grouping
	Olive Oil	Salts	Thiamine		
A	+	+	+ *	19.87 ± 1.73 **	a b
B	+	+	-	19.05 ± 1.70	a b c
C	+	-	+	23.39 ± 2.44	a
D	+	-	-	14.24 ± 1.24	c d
E	-	+	+	12.23 ± 1.39	d
F	-	+	-	14.30 ± 0.98	c d
G	-	-	+	16.04 ± 0.94	b c d
H	-	-	-	12.56 ± 2.61	d

* “+” included and “-” not included; ** Standard deviations for n = 3.

**Table 3:** Analysis of variance calculated by Minitab 19 for the factorial design.

Source	DF *	Adj SS **	Adj MS ***	F-Value	*p*-Value
Model	7	327.497	46.785	15.60	0.000
Linear	3	220.794	73.598	24.54	0.000
Olive Oil	1	171.949	171.949	57.33	0.000
Salts	1	0.224	0.224	0.07	0.788
Thiamine	1	48.621	48.621	16.21	0.001
2-Way Interactions	3	103.818	34.606	11.54	0.000
Olive Oil * Salts	1	4.234	4.234	1.41	0.252
Olive Oil * Thiamine	1	27.478	27.478	9.16	0.008
Salts * Thiamine	1	72.107	72.107	24.04	0.000
3-Way Interactions	1	2.884	2.884	0.96	0.341
Olive Oil * Salts * Thiamine	1	2.884	2.884	0.96	0.341
Error	16	47.992	3.000		
Total	23	375.489			

* DF: Degrees of freedom. ** Adj SS: Adjusted sum of squares. *** Adj MS: Adjusted mean square.

**Table 4:** Dry biomass concentration of each pair of treatments analyzed by Tukey’s method of multiple comparisons for the olive oil–thiamine interaction. n = 3.

Treatment Pairs	Level		Mean Dry Biomass Concentration (g/L)	Group		
	Olive Oil	Thiamine				
A-C	+	+	21.63 ± 2.70 *	a		
B-D	+	-	16.64 ± 2.95		b	
E-G	-	+	14.14 ± 2.34		b	c
F-H	-	-	13.43 ± 2.01			c

* s.d.

**Table 5:** Dry biomass concentration for each pair of treatments analyzed by Tukey’s multiple comparison test for the salt–thiamine interaction, n = 3.

Means of Treatments	Concentration		Mean Dry Biomass Concentration (g/L)	Group		
	Salts	Thiamine				
C-G	-	+	19.71 ± 4.35 *	a		
B-F	+	-	16.67 ± 2.88		b	
A-E	+	+	16.05 ± 4.41		b	c
D-H	-	-	13.40 ± 2.05			c

* s.d.

**Table 6:** Specific growth rate of *Ganoderma* sp. in the submerged culture obtained from different growth models.

Model	Specific Growth Rate (h^−1^)	Standard Error of the Regression (g/L)	AICc
Exponential	0.0041 ± 0.0005 *	2.42	42.54
Logistic	0.0139 ± 0.0026	1.76	28.54
Gompertz	0.0087 ± 0.0019	1.67	25.93
Richards	0.0087 ± 0.0108	1.71	28.56

* Standard error for the parameter estimation.

**Table 7:** Reported dry biomass concentrations and culture conditions for *Ganoderma* sp. growth in stirred tank bioreactors.

Culture Medium (g/L)	Operating Conditions	Bioreactor Configuration	Operation Mode	Dry Biomass Concentration (g/L)	Ref.
Glc 30, Pep 5, YE 5, KH_2_PO_4_ 0.5, K_2_HPO_4_ 0.5, MgSO_4_ 7H_2_O 0.5, B1 0.05, OO 9.1	30 °C, pH 5.5, 0.5 g/L, 12 d	2 L, 350 rpm, 0.25 vvm, MA-PI6-RT, PS	Batch	22.6 (12 d)	This work
Glc 16, Pep 2.93, CF 20.93, SBP 6.44, KH_2_PO_4_ 1.5, MgSO_4_ 7H_2_O 1	30 °C, 2.0 g/L, 6 d	35 L, 125 rpm, 0.6 vvm	Batch	21.5 (5 d)	[18]
Glc 55, YE 14.3, KH_2_PO_4_ 1, MgSO_4_ 7H_2_O 0.26, Fe_2_(SO_4_)_3_ 0.34, B1 0.05	30 °C, pH 5.5, 0.5 g/L, 12 d	7 L, 300 rpm, 1.0 vvm, RT-RT, RS	Batch	25.7 (12 d)	[19]
WB 200, YE 80	30 °C, pH 6.0, 14.7 g/L, 8 d	4 L, 200 rpm, 1.0 vvm, RT	Batch	28.2 (8 d)	[20]
Lac 35, Pep 5, YE 5, KH_2_PO_4_ 1, MgSO_4_ 7H_2_O 0.5, B1 0.05	30 °C, pH 5.5, pO_2_ 20–35%, 0.6 g/L, 22 d	2 L, 100–180 rpm, 0.25–0.5 vvm, RT-RT, RS	Fed batch	21.9 (12 d)	[21]
Glc 35, Pep 5, YE 5, KH_2_PO_4_ 1, MgSO_4_ 7H_2_O 0.5	30 °C, pH 4.0, pO_2_ 20%, 0.5 g/L, 10 d	10 L, 300 rpm, 2.0 vvm (max)	Fed batch	26.6 (10 d)	[22]
Lac 35, Pep 5, YE 5, KH_2_PO_4_ 1, MgSO_4_ 7H_2_O 0.5, B1 0.05	30 °C, pH 3.0–4.5, pO_2_ 25–10%, 0.6 g/L, 18 d	5.5 L, 50–400 rpm, 0.1–0.7 vvm, RT-RT-PI4, RS	Fed batch	22.6 (12 d)	[23]
Glc 25, Suc 20, YE 14, KH_2_PO_4_ 1, MgSO_4_ 7H_2_O 0.26, B1 0.05	30 °C, pH 5.5, 0.5 g/L, 10 d	7 L, 300 rpm, 1.0 vvm	Fed batch	29.7 (9 d)	[24]

## Data Availability

Data is contained within the article.

## References

[1] Legarda L.X., Echevarria A.C., Sánchez S.F. (2015). Producción de polisacáridos a partir de *Ganoderma* sp., aislado en la región andina. Rev. Colomb. Biotecnol..

[2] López-Peña D., Samaniego-Rubiano C., Morales-Estrada I., Gutiérrez A., Gaitán-Hernández R., Esqueda M. (2019). Características morfológicas de Ganoderma subincrustatum silvestre y cultivada de Sonora, México. Sci. Fungorum.

[3] Ruiz-Boyer A. The family Ganodermataceae (Aphyllophorales) in Costa Rica. La familia Ganodermataceae (Aphyllophorales) en Costa Rica. Tropical Diversity Origins, Maintenance, and Conservation. Proceedings of the ATB & OTS Symposium and Annual Meeting Abstracts.

[4] Ruiz-Boyer A., Rodríguez-González A. (2020). Lista preliminar de hongos (Ascomycota y Basidiomycota) y mixomicetos (Myxomycota) de la Isla del Coco, Puntarenas, Costa Rica. Rev. Biol. Trop..

[5] Cid-Martínez M.A., Gallardo-Velázquez K., Rosique-Gil J.E., Domínguez-Rodríguez V.I., Focil-Monterrubio R.L. (2019). Cuantificación de las esporas de ganoderma del aire exterior en la ciudad de Villahermosa, Tabasco, México. Rev. Int. Contam. Ambient..

[6] Lu J., He R., Sun P., Zhang F., Linhardt R.J., Zhang A. (2020). Molecular mechanisms of bioactive polysaccharides from Ganoderma lucidum (Lingzhi), a review. Int. J. Biol. Macromol..

[7] Soccol C.R., Bissoqui L.Y., Rodrigues C., Rubel R., Sella S.R., Leifa F., Soccol V.T. (2016). Pharmacological properties of biocompounds from spores of the lingzhi or reishi medicinal mushroom Ganoderma lucidum (Agaricomycetes): A review. Int. J. Med. Mushrooms.

[8] Soylu E.M., Soylu S., Kurt S. (2006). Antimicrobial Activities of the Essential Oils of Various Plants against Tomato Late Blight Disease Agent Phytophthora infestans. Mycopathologia.

[9] Yang F.-C., Ke Y.-F., Kuo S.-S. (2000). Effect of fatty acids on the mycelial growth and polysaccharide formation by Ganoderma lucidum in shake flask cultures. Enzym. Microb. Technol..

[10] Xu Y.-N., Zhong J.-J. (2012). Impacts of calcium signal transduction on the fermentation production of antitumor ganoderic acids by medicinal mushroom Ganoderma lucidum. Biotechnol. Adv..

[11] Tang Y.-J., Zhu L.-W. (2010). Improvement of Ganoderic Acid and Ganoderma Polysaccharide Biosynthesis by Ganoderma lucidum Fermentation Under the Inducement of Cu^2+^. Biotechnol. Prog..

[12] Schnellbaecher A., Binder D., Bellmaine S., Zimmer A. (2019). Vitamins in cell culture medium: Stability and stabilization strategies. Biotechnol. Bioeng..

[13] Zárate-Chaves C.A., Romero-Rodríguez M.C., Niño-Arias F.C., Robles-Camargo J., Linares-Linares M., Rodríguez-Bocanegra M.X., Gutiérrez-Rojas I. (2013). Optimizing a culture medium for biomass and phenolic compounds production using Ganoderma lucidum. Braz. J. Microbiol..

[14] Wagner R., Mitchell D.A., Sassaki G., Lopes de Almeida Amazonas M.A., Berovi M. (2003). Current Techniques for the Cultivation of Ganoderma lucidum for the Production of Biomass, Ganoderic Acid and Polysaccharides. Food Technol. Biotechnol..

[15] Fang Q.-H., Tang Y.-J., Zhong J.-J. (2002). Significance of inoculation density control in production of polysaccharide and ganoderic acid by submerged culture of Ganoderma lucidum. Process. Biochem..

[16] Chang M.-Y., Tsai G.-J., Houng J.-Y. (2006). Optimization of the medium composition for the submerged culture of Ganoderma lucidum by Taguchi array design and steepest ascent method. Enzym. Microb. Technol..

[17] Tang Y.-J., Zhong J.-J. (2003). Role of oxygen supply in submerged fermentation of Ganoderma lucidum for production of Ganoderma polysaccharide and ganoderic acid. Enzym. Microb. Technol..

[18] Jo W.-S., Cho Y.-J., Cho D.-H., Park S.-D., Yoo Y.-B., Seok S.-J. (2009). Culture Conditions for the Mycelial Growth ofGanoderma applanatum. Mycobiology.

[19] Feng J., Zhang J.-Z., Feng N., Yan M.-Q., Yang Y., Jia W., Lin C.-C. (2017). A novel Ganoderma lucidum G0119 fermentation strategy for enhanced triterpenes production by statistical process optimization and addition of oleic acid. Eng. Life Sci..

[20] Spiess A.-N., Neumeyer N. (2010). An evaluation of R2 as an inadequate measure for nonlinear models in pharmacological and biochemical research: A Monte Carlo approach. BMC Pharmacol..

[21] Kim H.M., Kim S.W., Hwang H.J., Park M.K., Mahmoud Y.A.-G., Choi J.W., Yun J.W. (2006). Influence of Agitation Intensity and Aeration Rate on Production of Antioxidative Exopolysaccharides from Submerged Mycelial Culture of Ganoderma resinaceum. J. Microbiol. Biotechnol..

[22] Salmon D.N., Fendrich R.C., Cruz M.A., Weingartner-Montibeller V., Vandenberghe L.P., Soccol C.R., Rigon-Spier M. (2016). Bioprocess for phytase production by Ganoderma sp. MR-56 in different types of bioreactors through submerged cultivation. Biochem. Eng. J..

[23] Tang Y.-J., Zhong J.-J. (2002). Fed-batch fermentation of Ganoderma lucidum for hyperproduction of polysaccharide and ganoderic acid. Enzym. Microb. Technol..

[24] Lejeune R., Baron G.V. (1998). Modeling the exponential growth of filamentous fungi during batch cultivation. Biotechnol. Bioeng..

[25] Yorulmaz A., Erinc H., Tekin A. (2013). Changes in Olive and Olive Oil Characteristics During Maturation. J. Am. Oil Chem. Soc..

[26] Lv G.-P., Zhao J., Duan J.-A., Tang Y.-P., Li S.-P. (2012). Comparison of sterols and fatty acids in two species of Ganoderma. Chem. Cent. J..

[27] Fei Y., Li N., Zhang D.-H., Xu J.-W. (2019). Increased production of ganoderic acids by overexpression of homologous farnesyl diphosphate synthase and kinetic modeling of ganoderic acid production in Ganoderma lucidum. Microb. Cell Factor..

[28] Wei Z.-H., Duan Y.-Y., Qian Y.-Q., Guo X.-F., Li Y.-J., Jin S.-H., Zhou Z.-X., Shan S.-Y., Wang C.-R., Chen X.-J. (2014). Screening of Ganoderma strains with high polysaccharides and ganoderic acid contents and optimization of the fermentation medium by statistical methods. Bioproc. Biosyst. Eng..

[29] Torres-López A.M., Quintero-Díaz J.C., Atehortúa-Garcés L. (2011). Efecto de nutrientes sobre la producción de biomasa del hongo medicinal Ganoderma lucidum. Rev. Colomb. De Biotecnol..

[30] Papagianni M. (2004). Fungal morphology and metabolite production in submerged mycelial processes. Biotechnol. Adv..

[31] Guevara-Manzanares V. (2015). Producción de Ácidos Ganodéricos y Beta-(1–3)-(1–6)-D-Glucanos en el Cultivo Líquido Sumergido de Ganoderma sp. de Interés Medicinal en la Industria Alimenticia de Costa Rica.

